# Supplementary figures and images for: OrBITS: label-free and time-lapse monitoring of patient derived organoids for advanced drug screening
Source: Cell Oncol (Dordr). 2022 Dec 12;46(2):299–314. doi: 10.1007/s13402-022-00750-0 (PMC10060271; doi:10.1007/s13402-022-00750-0)

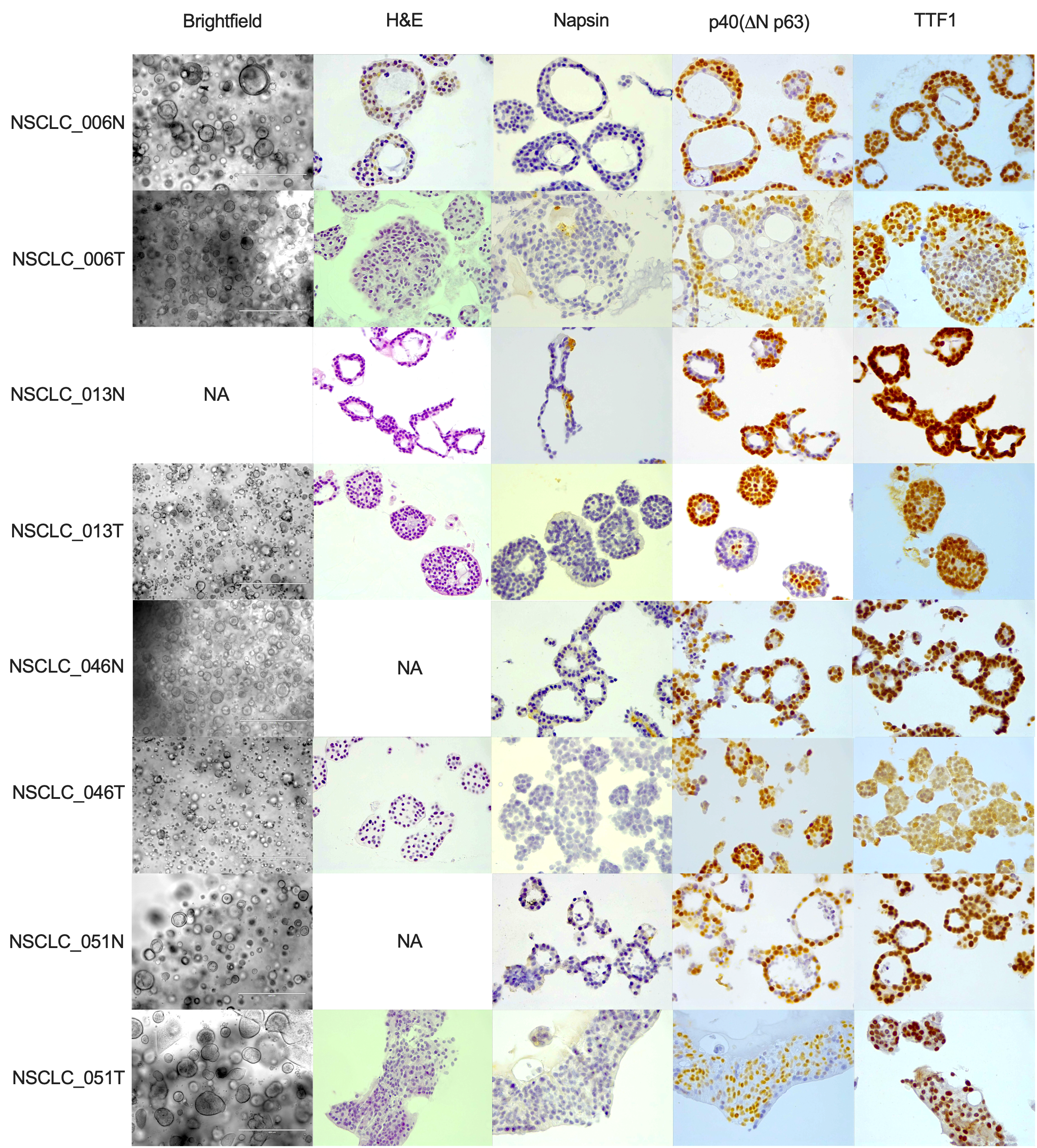

Supplement: Supplementary file 8 — Supplementary Material 8 [file 13402_2022_750_MOESM8_ESM.png]

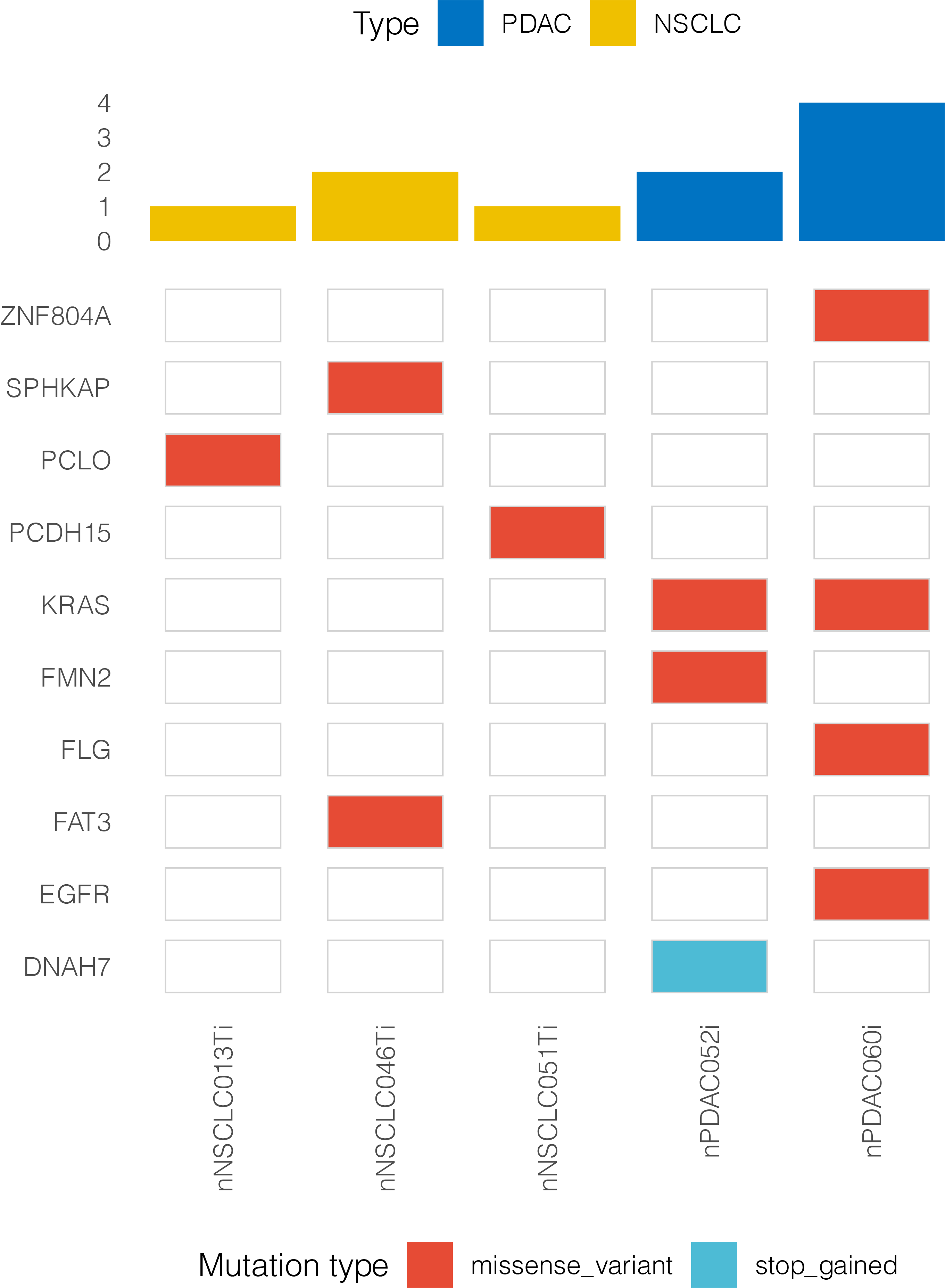

Supplement: Supplementary file 9 — Supplementary Material 9 [file 13402_2022_750_MOESM9_ESM.png]

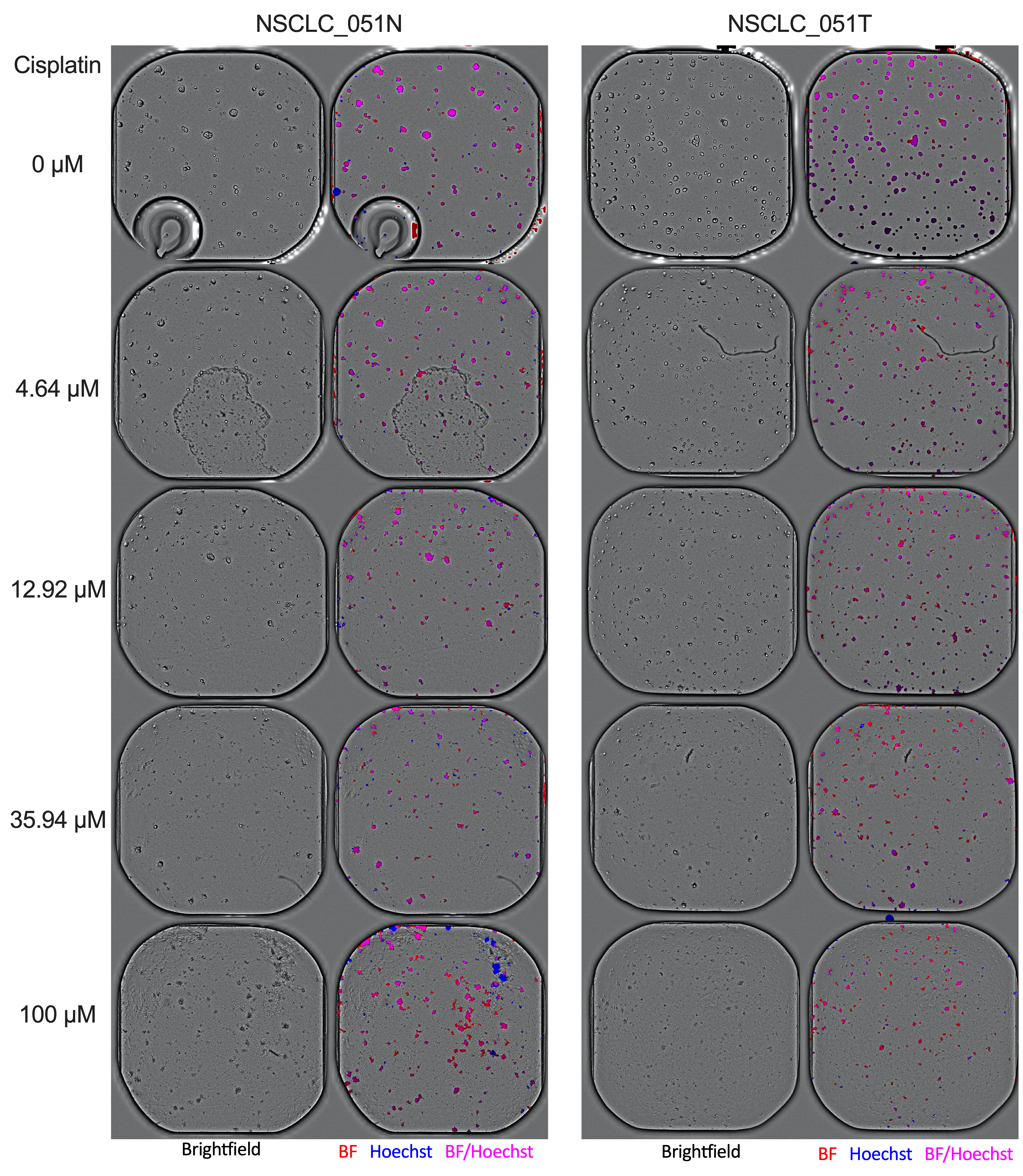

Supplement: Supplementary file 10 — Supplementary Material 10 [file 13402_2022_750_MOESM10_ESM.png]

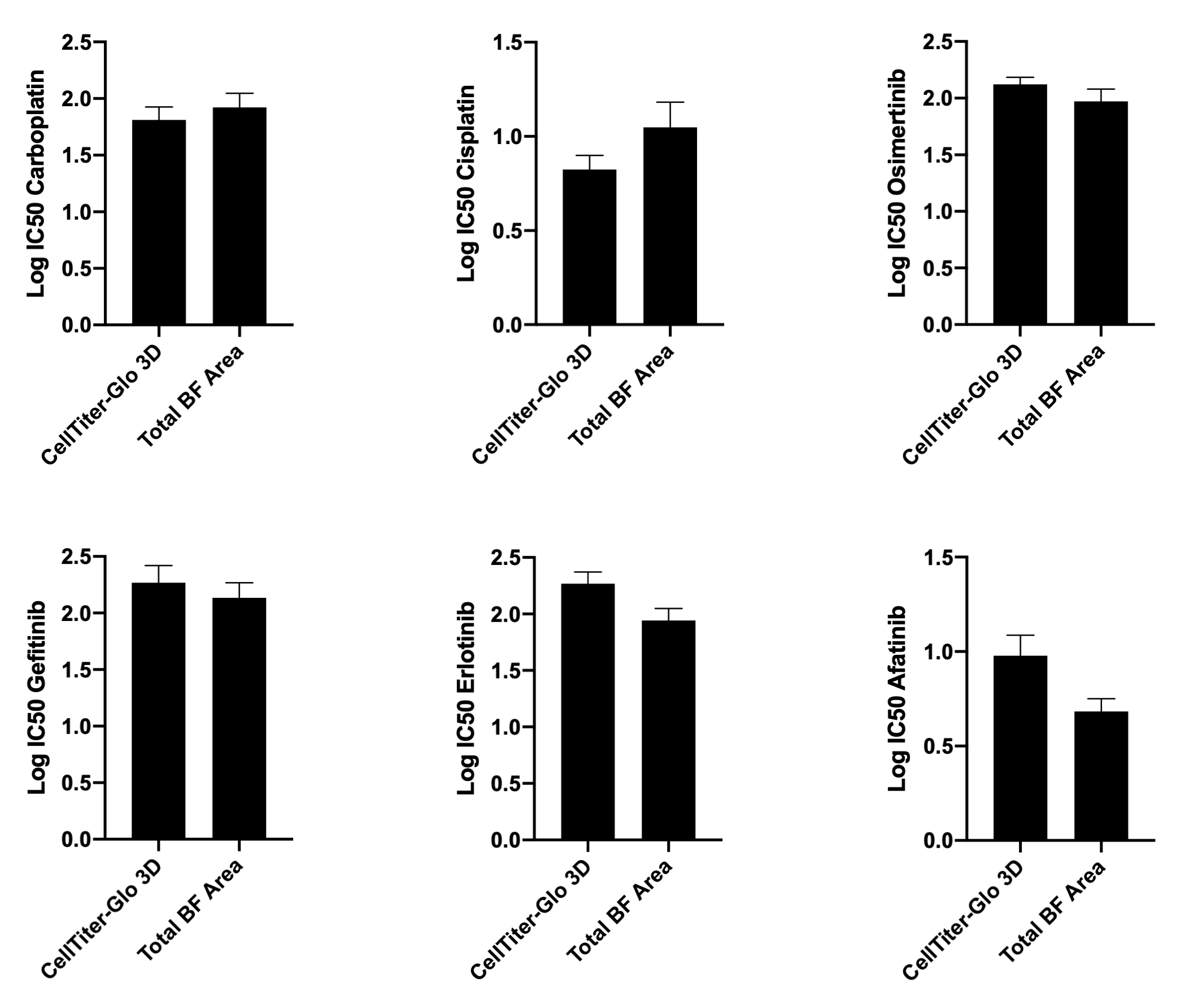

Supplement: Supplementary file 11 — Supplementary Material 11 [file 13402_2022_750_MOESM11_ESM.png]
